# Genomic traits of *Klebsiella oxytoca* DSM 29614, an uncommon metal-nanoparticle producer strain isolated from acid mine drainages

**DOI:** 10.1186/s12866-018-1330-5

**Published:** 2018-11-27

**Authors:** Giuseppe Gallo, Luana Presta, Elena Perrin, Michele Gallo, Davide Marchetto, Anna Maria Puglia, Renato Fani, Franco Baldi

**Affiliations:** 1 Laboratory of Molecular Microbiology and Biotechnology, Department of Biological, Chemical and Pharmaceutical Sciences and Technologies, Viale delle Scienze, ed. 16, 90128 Palermo, Italy; 2 Laboratory of Microbial and Molecular Evolution, Department of Biology, University of Florence, Via Madonna del Piano 6, I-50019 Sesto F.no, Florence, Italy; 3 Dipartimento di Scienze Molecolari e Nanosistemi, University Cà Foscari Venezia, Via Torino 155, 30172 Mestre, Venezia Italy

**Keywords:** Genome, Capsular exopolysaccharide, Ferric-hydroxide gel, Iron, Metal resistance, Metal nanoparticles

## Abstract

**Background:**

*Klebsiella oxytoca* DSM 29614 - isolated from acid mine drainages - grows anaerobically using Fe(III)-citrate as sole carbon and energy source, unlike other enterobacteria and *K. oxytoca* clinical isolates. The DSM 29614 strain is multi metal resistant and produces metal nanoparticles that are embedded in its very peculiar capsular exopolysaccharide. These metal nanoparticles were effective as antimicrobial and anticancer compounds, chemical catalysts and nano-fertilizers.

**Results:**

The DSM 29614 strain genome was sequenced and analysed by a combination of in silico procedures. Comparative genomics, performed between 85 *K. oxytoca* representatives and *K. oxytoca* DSM 29614, revealed that this bacterial group has an *open* pangenome, characterized by a very small *core* genome (1009 genes, about 2%), a high fraction of unique (43,808 genes, about 87%) and accessory genes (5559 genes, about 11%). Proteins belonging to COG categories “Carbohydrate transport and metabolism” (G), “Amino acid transport and metabolism” (E), “Coenzyme transport and metabolism” (H), “Inorganic ion transport and metabolism” (P), and “membrane biogenesis-related proteins” (M) are particularly abundant in the predicted proteome of DSM 29614 strain. The results of a protein functional enrichment analysis - based on a previous proteomic analysis – revealed metabolic optimization during Fe(III)-citrate anaerobic utilization. In this growth condition, the observed high levels of Fe(II) may be due to different flavin metal reductases and siderophores as inferred form genome analysis. The presence of genes responsible for the synthesis of exopolysaccharide and for the tolerance to heavy metals was highlighted too. The inferred genomic insights were confirmed by a set of phenotypic tests showing specific metabolic capability in terms of i) Fe^2+^ and exopolysaccharide production and ii) phosphatase activity involved in precipitation of metal ion-phosphate salts.

**Conclusion:**

The *K. oxytoca* DSM 29614 unique capabilities of using Fe(III)-citrate as sole carbon and energy source in anaerobiosis and tolerating diverse metals coincides with the presence at the genomic level of specific genes that can support i) energy metabolism optimization, ii) cell protection by the biosynthesis of a peculiar exopolysaccharide armour entrapping metal ions and iii) general and metal-specific detoxifying activities by different proteins and metabolites.

**Electronic supplementary material:**

The online version of this article (10.1186/s12866-018-1330-5) contains supplementary material, which is available to authorized users.

## Background

The genus *Klebsiella* embeds different Gram-negative rod shaped bacteria including human pathogens [1], harmless nitrogen–fixing plant endosymbionts [2], and polluted soil inhabitants [3, 4]. *Klebsiella oxytoca* (formerly known as *Klebsiella pneumoniae* or *Aerobacter aerogenes*) is a free-living bacterium isolated from soils [3], plants and animals, including humans. *K. oxytoca* strains can be either pathogenic [5] or beneficial having roles, for instance, as plant growth promoters [3, 6] and as valuable biochemical-compound producers [7].

The strain *K. oxytoca* DSM 29614 (ex BAS-10) was isolated under an iron hydroxides mat from an acid drainage of a pyrite mine at Metalliferous Hills in Southern Tuscany, Italy [8, 9]. Interestingly, this strain is able to grow using Fe(III)-citrate as sole carbon source under anaerobic condition, producing Fe(II) from Fe(III) and a peculiar exopolysaccharide (EPS) containing rhamnose, glucuronic acid and galactose [10, 11]. The EPS is produced also in the presence of toxic heavy metals, such as Cd^2+^, Pb^2+^ and Zn^2+^ [12], As^+ 5^ and As^+ 3^ [13], and Hg^2+^ [14]. In such conditions, metals often precipitate with EPS in the form of metal nanoparticles embedded in the EPS polymer. This biotechnological peculiarity was used to produce biogenerated “green” metal nanoparticles (NPs) containing: Fe [15, 16], Pd, and Rh that were used for catalytic reactions [17–19]; bimetallic Fe-Pd clusters that were used for hydrodechlorination of chlorobenzene [20] and Aroclor 1260 [21]; Ag that exerts antimicrobial [22, 23] and anticancer [24] activities. Recently, the Fe(III)-EPS was used as a nutraceutical compound in order to improve growth rate of the commercial fungus *Tuber borchii* by improving Fe^3+^ uptake [25]. Proteomic analysis performed by 2D-Differential Gel Electrophoresis (2D-DIGE) and mass spectrometry (MS) procedures revealed that protein differential regulation seems to ensure efficient cell growth coupled with EPS production by adapting metabolic and biochemical processes in order to face iron toxicity and to optimize energy production [26]. Altogether, these characteristics prove insights into the remarkable biotechnological usefulness of this bacterium.

Given such an encouraging picture, the aim of this work was to investigate the *K. oxytoca* DSM 29614 genetic features accounting for the synthesis of EPS and heavy metal resistance conferring, thus, the capability of diverse metal nanoparticle (NP) production. To this scope, the *K. oxytoca* DSM 29614 genome was sequenced and analysed by a combination of in silico procedures. Moreover, a set of phenotypic tests was carried out to corroborate the achieved genomic insights.

## Methods

### Culture conditions

Cells of *K. oxytoca* DSM 29614 (ex BAS-10) strain were stored in cryovials at − 80 °C in 25% glycerol solution until they were retrieved using Difco Nutrient Broth (BD Bioscience, Italy) growth medium at 30 °C. Aerobic and anaerobic cultivations were performed as described in Buttacavoli et al. (2018) [24].

When the strain was grown in the presence of heavy metals either the NaC or FeC media were used. The NaC medium contains per liter of distilled water: 2.5 g NaHCO_3_, 1.5 g NH_4_Cl, 1.5 g MgSO_4_.7H_2_O, 0.6 g NaH_2_PO_4_, 0.1 g KCl, and 14.7 g Na(I)-citrate (Carlo Erba, Italy). The NaC medium was buffered at pH 7.8 with a solution of NaOH. In the FeC medium 50 mM Fe(III)-citrate replaces 50 mM Na-citrate [27]*.* The growth of the strain was monitored determining total protein content by using Protein Assay kit (Bio-Rad, Italy), based on Bradford dye-binding method (1976) [28], according to manufacturing instruction for micro-assay.

### DNA isolation and whole genome sequencing

*K. oxytoca* DSM 29614 strain was grown at 30 °C using Difco Nutrient Broth (BD Bioscience, Italy) growth medium. The genomic DNA was extracted using the CTAB method [29] and the authenticity of the genomic DNA was confirmed by 16S rRNA gene sequencing (Ylichron Srl, Italy). Whole genome shot-gun sequencing was performed by using the Illumina HiSeq system (Illumina Inc., San Diego, CA) with a paired-end approach loading genomic DNA onto one single flowcell (BGI-HongKong Co. limited, Hong Kong).

### Phylogenetic analysis

16S rRNA gene sequences from strains belonging to *K. oxytoca* were selected from NCBI ftp site to run a phylogenetic analysis. Sequence alignment and phylogenetic tree were obtained as previously described in Presta et al. (2017) [30].

### Genome assembly and annotation

Poor quality bases were removed using the dynamic trimming algorithm embedded in the SolexaQA suite [31] selecting a Phred score threshold value of 13. Assembly was performed by using ABySS 1.3.7 software [32]. The resulting contigs were launched in a multidraft-based scaffolder MeDuSa [33] which starting from 307 initial contigs generated 62 scaffolds. The last ones were then annotated by using NCBI Automated Genome Annotation Pipeline.

### Comparative genomics

All the 85 *K. oxytoca* representative (Additional file 1: Table S1) genomes sequenced up to date (i.e. September 2016) were collected from the NCBI ftp site (ftp://ftp.ncbi.nlm.nih.gov/genomes/refseq/bacteria/Klebsiella_oxytoca/all_assembly_versions/) and, alongside *K. oxytoca* DSM 29614 genome, they were analyzed using Roary software [34] to identify shared orthologous and strain-specific genes.

### Identification of genes putatively involved in metal tolerance, secondary metabolite biosynthesis and antibiotic resistance

AntiSMASH software [35] for genome-wide identification, annotation and analysis of secondary metabolite biosynthetic gene clusters was used to scan the genome sequence of *K. oxytoca* DSM 29614 in order to identify gene clusters involved in secondary metabolite production*.*

BacMet database [36] was used to identify the genetic elements responsible for heavy metal resistance, by using BLASTp algorithm with a threshold e-value ≤0.0001 and percentage identity ≥30.

### Protein functional enrichment analysis

**Fig. 1:**
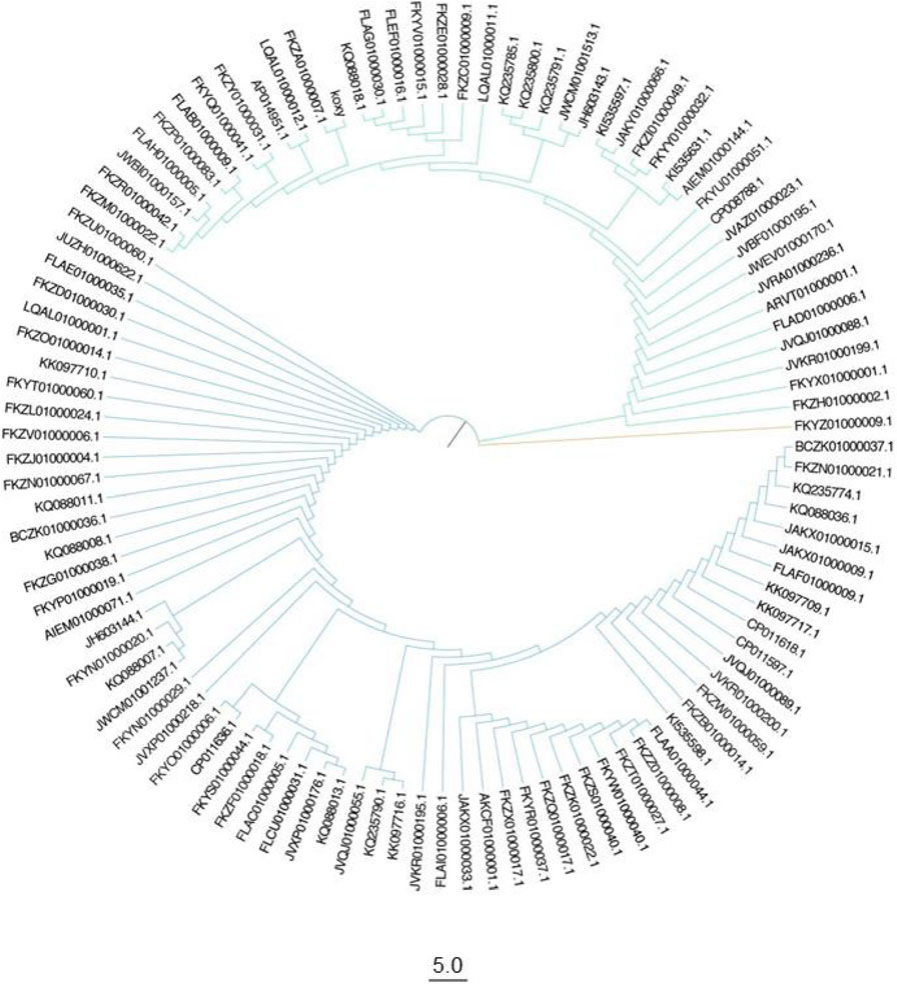
Phylogenetic tree based on 16S rRNA gene sequences of K. oxytoca representatives. *K. oxytoca* DSM 29614 is reported as koxy

Protein functional enrichment analysis was performed using the SmartTables tool at BioCyc database collection [37] with the option “Fisher Exact Parent-Child Union” (FEPCU) on. Two analyses were performed on the sets of differentially abundant proteins (Additional file 2: Table S2) obtained from the proteomic analyses described in Gallo et al. (2012) [26]. In particular, up-regulated proteins i) in FeC versus NaC in anaerobic condition and ii) in anaerobic versus aerobic FeC cultivations were used. For these analyses, the *K. oxytoca* 10–5243 (NCBI accession JH603143.1) was chosen as reference strain since it was the most related strain among the selectable ones at BioCyc database collection according to the phylogenetic tree based on *K. oxytoca* 16S rRNA gene sequences (Fig. 1).

### EPS extraction and determination

Aliquots of 10 ml of of DSM 29614 anaerobic culture in FeC medium were sampled at different times. The samples were firstly centrifuged to eliminate bacterial cells, then precipitation of polysaccharides was induced by treating the supernatant with 8 ml of cooled ethyl alcohol (95%). The purification phase was repeated twice. The concentration of total carbohydrate in EPS was quantified spectrophotometrically (485 nm) as glucose (Fluka, Germany) equivalents after reaction with 96% sulfuric acid (Fluka, Germany) and 5% phenol (Fluka, Germany) [38]. Iron removal from polysaccharide was performed by treating the rusty gel with 100 mmol l^−1^EDTA (Fluka, Germany) until it was colorless [10].

### Determination of total iron and Fe(II)

In order to quantify iron species in *K. oxytoca* DSM 29614 cultivations, 3-ml aliquots were collected at different times and processed for colorimetric assay as previously described in Baldi et al. (2009) [10].

### Struvite formation and determination

A 100 ml of NaC medium was inoculated with 1 ml of *K. oxytoca* DSM 29614 (OD_600nm_ = 0 1 ABS) and after 3 h of incubation at 30 °C, each culture was amended with 5 mg of Ag(NO_3_), Hg(NO_3_)_2_ or Pd(NO_3_)_2_ (Sigma-aldrich, Germany). The cultures were incubated at 30 °C in static mode until colloidal material and the crystals precipitated. Crystals were easily separated by filtration from colloidal fraction and the determination of crystal was performed with X-ray-Diffractometry (Bruker D8, Italy) with 40 kV and 40 mA geometry with Cu Kα (λ = 0.154 nm) radiation. The micro-crystals formed in the cultures added with Pd^2+^ were observed by optical microscopy (Axio-Imager Z1stand, Zeiss, Italy) equipped with Axiocam MRm 2.8 in transmission and in fluorescence modes. The specimens were stained with DAPI (Fluka, Germany) to observe cells involved in crystal precipitation.

### TEM observation of struvite

A Pd-EPS aliquot of 1 mg was suspended in 1 ml of milli-Q water and sonicated for 10 min. Then, in order to obtain TEM images a 5 μl aliquot of each suspension was processed as previously described in Battistel et al. (2015) [23].

### Determination of metals in struvite

Total metals in 10 mg of crystals from *K.oxytoca* DSM 29614 culture was determined in triplicates. The sample was mineralized with aqua-regia for 4 h at 70 °C. The total metals were determined by flameless Atomic Absorption Spectroscopy (Varian SpectrAA 250 Plus, Italy).

### Determination of alkaline phosphatase activity

The *K. oxytoca* DSM 29614 cells were harvested from aerobic and anaerobic NaC and FeC media without metal additions and with additions of AgNO_3_, Hg(NO_3_)_2_ or Pd(NO_3_)_2_ (Sigma-aldrich, Germany). The cells were harvested by centrifuging, washed and then recovered in 5 ml of carbonate buffer constituted by 0.095 M NaHCO_3_, 0.005 M Na_2_CO_3_, 0.1 M NaCl, 0.05 M MgCl_2_, at pH 8.5.

Aliquots of 3 ml of cell suspension were lysed on ice bath by a Vibra-Cell VC50 sonicator (Sonics & Materials, Newtown, USA), equipped with a 3-mm microtip. Two cycles of 45 s of pulsed sonication (30 s of pulse and 15 s of pause) were performed for each sample. The sonicator was set to 20 kHz, with a power output of 50 W. Cytosol with phosphatase activity was collected after centrifugation (20 min. at 14,100 *g*). The supernatant was recovered, filtered (0.02 mm pore size) and stored at − 20 °C until phosphatase assay was performed [39]. The supernatant (cytosol) and the pellet (cell membranes) were characterized for total protein concentration by Coomassie Blue dye (Biorad, Italy) assay protocol [28].

The enzymatic activity was determined in the presence of different concentrations of the fluorescence substrate MUF-PO_4_ (Sigma-Aldrich, Germany) [40] in order to determine V_max_ and K_m_. The florescence intensity was measured by using a spectrofluorometer (Victor mod. X2, Perkin-Elmer). The fluorescence excitation (E_ex_) and emission (E_em_) wavelengths were set up at 380 and 480 nm, respectively. The calculation of phosphatase activity (PA) was performed within a range of MUF-PO_4_ between 10 to 1000 μM. The PA activity is expressed as “IU”, namely the enzyme activity unit, which transforms 1 μM per minute of substrate. Than the PA, reported as international unit (IU), is normalized to the cell biomass calculated as grams of proteins (g_PRT_).

## Results

### *K. oxytoca* DSM 29614 genome

The whole-genome shotgun project has been deposited at NCBI WGS database under the accession number MKCU00000000 and the version reported in this work was named MKCU01000000. The genome is 6,257,287 bp long with a GC content of 51.85%, similarly to other members of the same species. Other main features of the genome are reported in Table 1.

**Table 1:** Main features of *Klebsiella oxytoca* DSM 29614 genome

Features		n°
Genes	Total	5968
	Coding	5739
CDS	Total	5924
	Coding	5739
CRISPR Arrays		1
ncRNAs		7
tRNAs		34
rRNAs	Total	1 (5S); 2 (16S)
	Complete	1 (5S); 1 (16S)
	Partial	1 (16S)
Pseudo Genes	Total	185
	Ambigous residues	1
	Frameshifted	40
	Incomplete	143
	Internal stop	16
	Multiple problems	14

Comparative genomics analyses were performed between all the 85 previously named *K. oxytoca* representatives (Additional file 1: Table S1) and *K. oxytoca* DSM 29614; the phylogeny of the genus has been also investigated and the diversity and composition of the global gene repertoire has been studied through the pangenome approach analysis, as described in Materials and Methods.

**Fig. 2:**
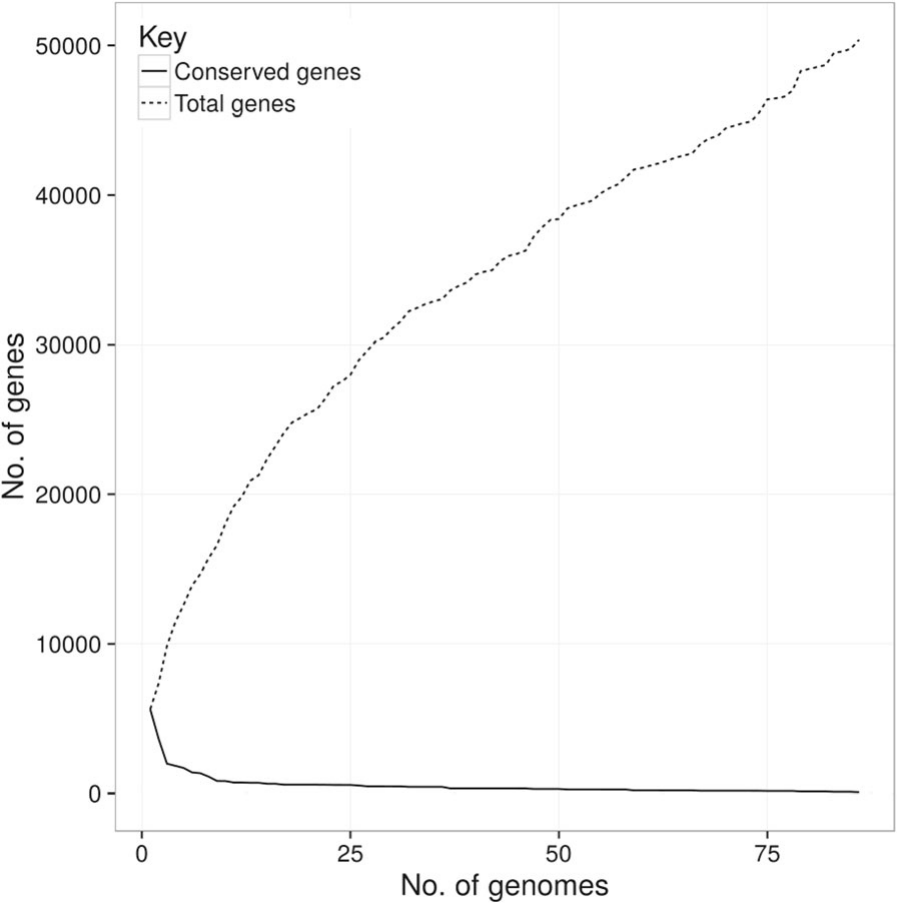
Diagram of conserved genes *per* number of genomes embedded into the analysis

**Fig. 3:**
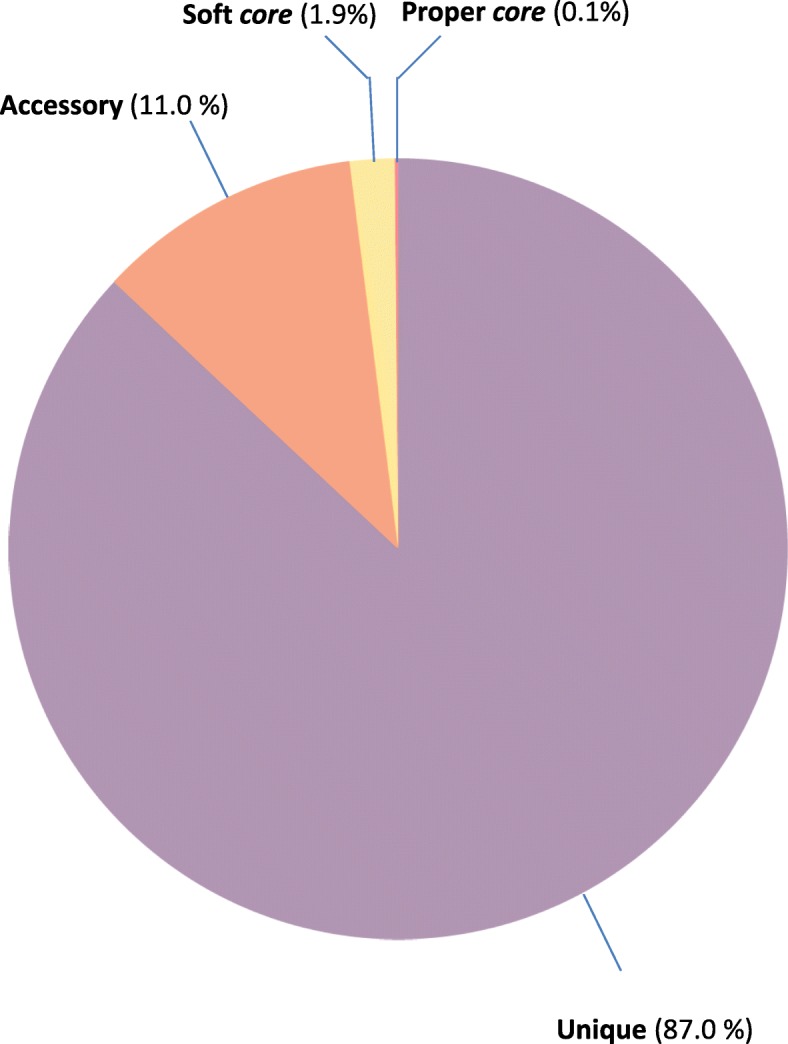
Diagram showing the percentage of *core*, soft-*core*, accessory, and unique genes of the *K. oxytoca* DSM 29164 genome

Despite the phylogenetic analysis revealed that the 16S rRNA coding sequences can be included into three main clusters (Fig 1), the phylogenetic history of these organisms appears to be very complex. In this regard, the data obtained by evaluating the number of conserved genes vs. the number of total genes (plotted in Fig. 2) revealed that this group of microorganisms has an *open* pangenome - i.e. the number of unique and accessory genes increases with the number of genomes embedded into the analysis. Moreover, Fig. 3 shows the size of *unique*, *accessory* and *core* genomes. The *core* genome, here presented as soft-*core* and proper *core*, consists of 1009 genes (about 2% of the total), whereas there is a high fraction of unique (43,808 genes, about 87%) and accessory genes (5559 genes, about 11%).

**Fig. 4:**
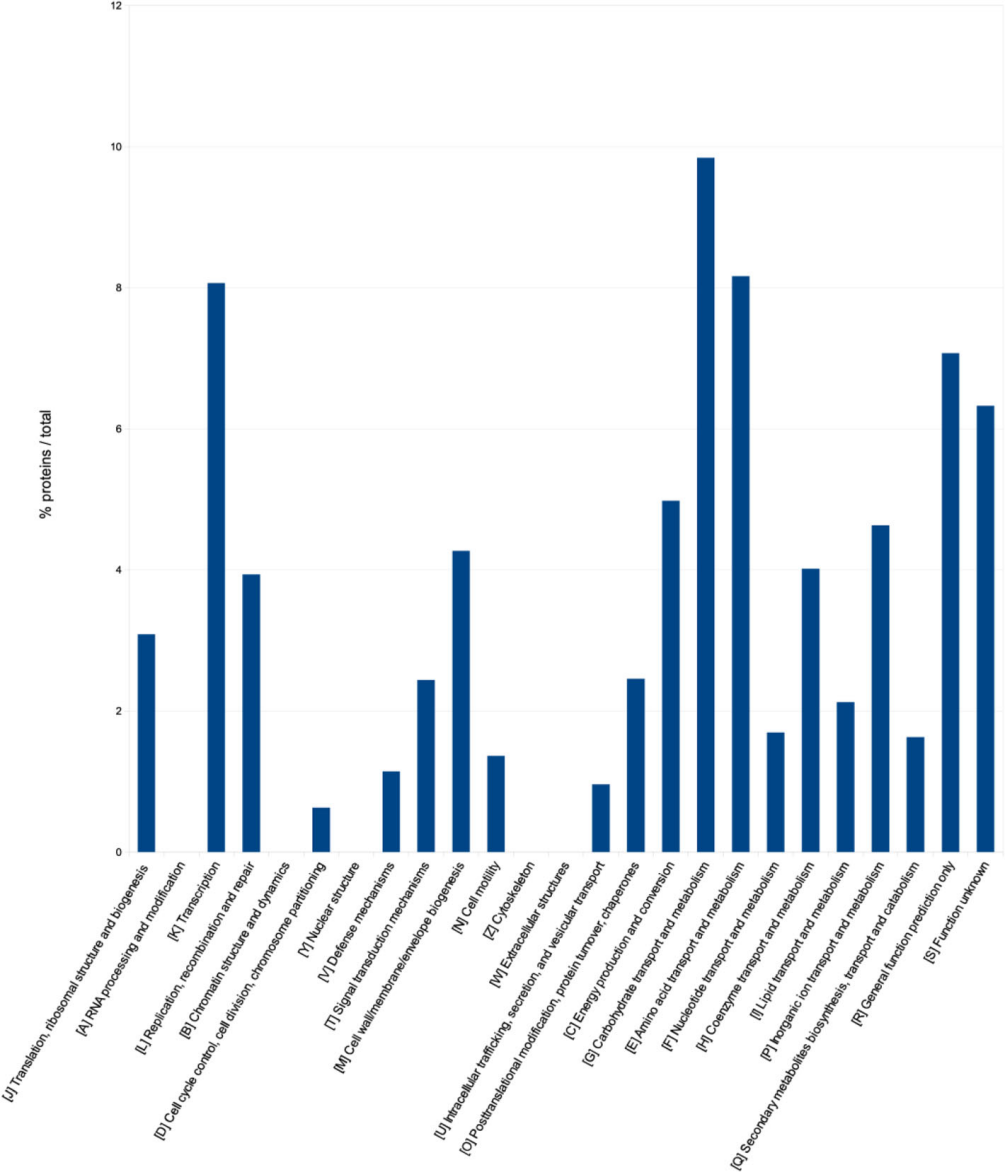
Proteins abundance *per* COG functional categories of *K. oxytoca* DSM 29614 genome

A deeper functional characterization of *K. oxytoca* DSM 29614 genome has been obtained by mapping its open-reading frames (ORFs) to the COG database [41]. Data obtained are shown in Fig. 4 reporting the abundance of genes assigned to each category. Such analysis revealed that proteins belonging to COG categories “Carbohydrate transport and metabolism” (G), “Amino acid transport and metabolism” (E), “Coenzyme transport and metabolism” (H), “Inorganic ion transport and metabolism” (P), and “membrane biogenesis-related proteins” (M) are particularly abundant in the predicted proteome.

### Insights from genomics for Fe(III)-citrate fermentation capability

**Fig. 5:**
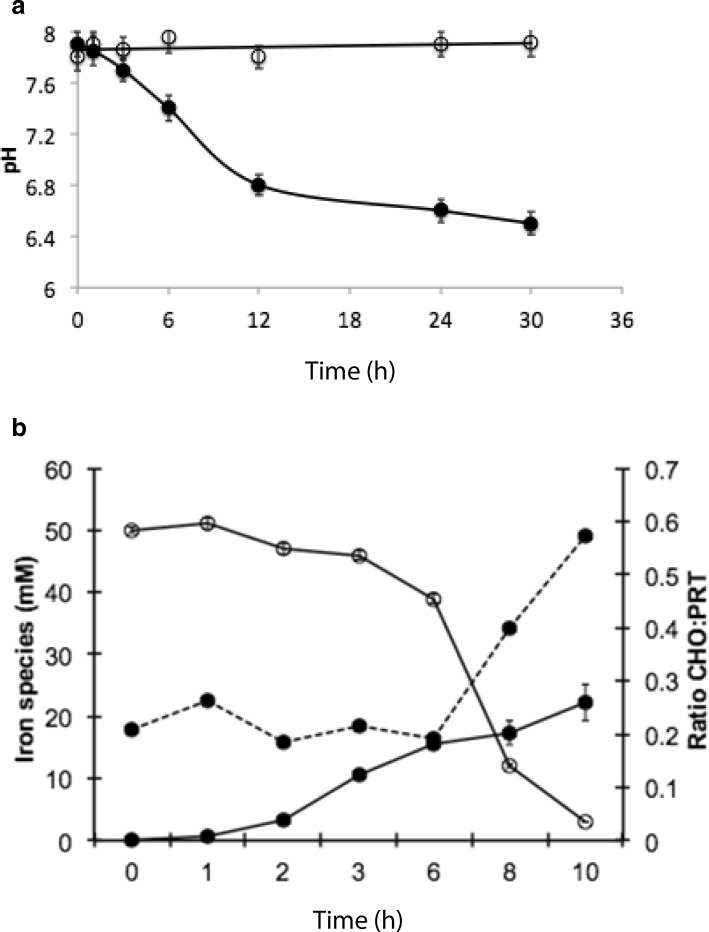
**a** Medium pH in *K. oxytoca* DSM 29614 cultures performed in FeC (empty circles) and in NaC (solid circles) medium under aerobic and anaerobc condition, respectively. **b** Determination of total Fe (empty circled), Fe^2+^ (solid circles) and ratio between polysaccharide (CHO) and proteins (PRT) of harvested DSM 29614 cells during anaerobic growth in FeC medium. Standard deviation values are reported as vertical bars

According to previous data [12], medium acidification occurs when DSM 29614 strain is incubated in presence of citrate under anaerobic conditions while medium pH is stable when the strain is incubated aerobically in presence of citrate (Fig. 5a). Indeed, the DSM 29614 strain genome contains the genes encoding citrate lyase (CitEF), Na^+^-dependent membrane oxaloacetate decarboxylase (OadA), pyruvate formate lyase (PFL), phosphotransacetylase (PTA) and acetate kinase (ACK), that are the enzymes required for anaerobic citrate utilization [42]. Interestingly, the differential representation of these enzymes was already described in two differentially abundant protein sets in Gallo et al. (2012) [26]. In the present work, these two protein sets (Additional file 2: Table S2) were used for a BioCyc-based functional enrichment analysis whose results are available at the internet address https://biocyc.org/smarttables as BioCyc Public Smart Tables “Enriched with FEPCU from anaerobic Vs aerobic growth on FeC up-regulated proteins” and “Enriched with FEPCU from FeC Vs NaC during anaerobic growth up-regulated proteins”. In particular, these results revealed an up-regulation of enzymes belonging to i) a super pathway including glycolysis, pyruvate dehydrogenase, TCA and glyoxylate bypass, ii) fermentation pathways including the one forming acetate, and iii) anaerobic respiration during anaerobic utilization of Fe(III)-citrate as carbon and energy source. Thus, this result further supports the capability of the DSM 29614 strain to optimize energy production.

**Fig. 6:**
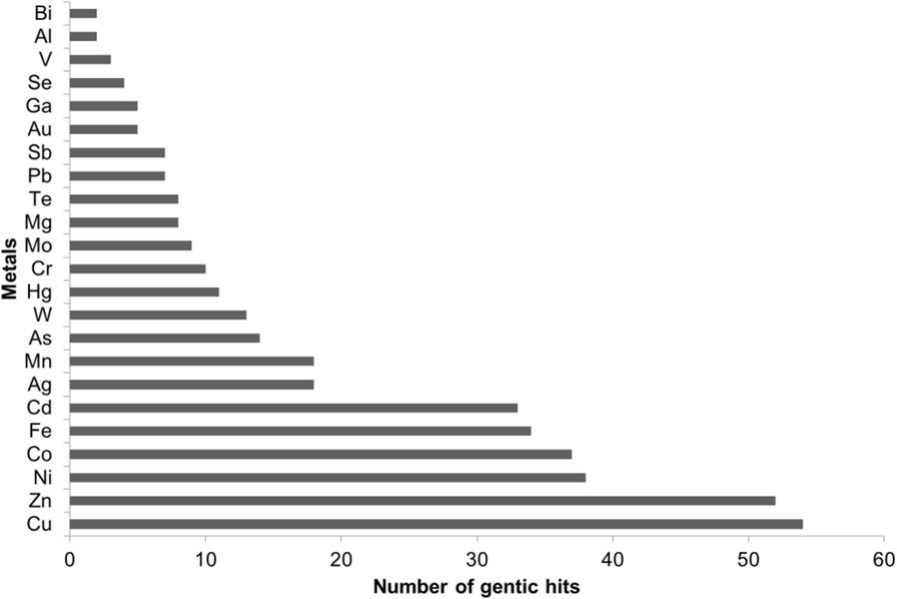
Number of genes, having homologues in *K. oxytoca* DSM 29614, putatively associated with tolerance to different metals according to BacMet database [36]

Another intriguing aspect of DSM 29614 strain is the massive reduction of 50 mM of Fe^3+^ to 22 mM Fe^2+^ during anaerobic growth on FeC medium (Fig. 5b). A BLAST interrogation revealed that the genome of DSM 29614 strain does not contain genes homologous to those found in *Shewanella* – like *cymA*, *mtrABC* operon, *mtrF* and *omcA -* or in *Geobacter* – such as *omcS*, *omcT*, *dhc2* and *pccF* - involved in dissimilatory Fe(III) reduction [43–46]. However, the DSM 29614 strain possesses genes that can have a role in iron transport and reduction. Among them there are: *fecABCDE* operon (BI322_RS14055- BI322_RS14075) that is devoted to Fe(III)-citrate uptake in bacterial cells [47]; genes encoding flavin metal reductases, like the flavodoxin FldA (BI322_RS16850), the NADPH-dependent ferric siderophore reductase (BI322_RS21400), the succinate dehydrogenase flavoprotein subunit (BI322_RS16715), and the flavocytochrome C (BI322_RS10335); genes encoding ion efflux pumps, such as the cation transporter FieF (BI322_RS11975) [45, 48]. In particular, the NADPH-dependent ferric siderophore reductase is one of the 33 gene products involved in iron tolerance predicted in DSM 29614 strain genome (Fig. 6; Additional file 3: Table S3), and both the succinate dehydrogenase flavoprotein subunit and the flavocytochrome C are predicted to play a role in the anaerobic respiration of fumarate according to KEGG database. Indeed, the succinate dehydrogenase flavoprotein subunit was upregulated in anaerobic FeC cultivation in the respect of both aerobic FeC cultivation and anaerobic NaC cultivation as previously revealed by differential proteomics [26].

In addition, the ability to produce bioactive compounds capable of acquiring Fe(III) was revealed by antiSMASH analysis showing the presence of a turnerbactin biosynthetic gene cluster (from BI322_RS17265 to BI322_RS17505). Indeed, turnerbactin is a triscatecholate siderophore synthetized through a Non Ribosomal Peptide Synthetase, which was firstly isolated from the shipworm endosymbiont *Teredinibacter turnerae* T7901 [49].

### Correlation between metal tolerance and genomic traits

A crucial contribution to tolerance to high levels of iron can be ascribed to the peculiar EPS produced by DSM 29614 strain which promotes metal ion entrapment and reduction. In fact, when the strain grows anaerobically in the presence of FeC, in concomitance to the Fe^3+^ reduction to Fe^2+^, iron also precipitates in the bacterial EPS (Fig. 5b; Additional file 4: Figure S1) [22]. In these growth conditions the pH is low and the citrate is consumed, but the increased ratio between polysaccharides (CHO) and cell proteins (PRT) in harvested cells at stationary phase demonstrated an overproduction of EPS (Fig. 5b).

Two *cps* gene clusters, namely *cps1* and *cps2* (Tables 2 and 3, respectively) were identified in the genome supporting the capability of *K. oxytoca* DSM 29614 cells to synthesize the EPS. Indeed, some features of *cps1* and *cps2*, including their corresponding flanking regions in the genome, highlight peculiar characteristics of *K. oxytoca* DSM 29614 as described in (Additional file 5: Supporting Information).

**Table 2:** The *cps1* gene cluster of *K. oxytoca* DSM 29614

Gene (Locus tag)	Start/Stop nucleotides	Encoded Protein			
		id	Function	% of Identity^a^	Comment
BI322_RS06885	549463/550359	WP_024360046.1	UTP-glucose-1-phosphate uridylyltransferase (GalF)	99	
BI322_RS06890	550820/551449	WP_082236967.1	acid phosphatase (phosphatidic acid phosphatase)	98	
BI322_RS06895	552089/552316	WP_082236968.1	hypothetical protein	0	
BI322_RS06900	552447/553886	WP_082236969.1	capsule assembly Wzi family protein (Wzi)	99	
BI322_RS06905	553960/555096	WP_082236970.1	polysaccharide export protein (Wza)	94	Wza is required for the translocation of capsular polysaccharide through the outer membrane.
BI322_RS06910	555098/555538	WP_082236971.1	protein tyrosine phosphatase (Wzb)	88	Wzb shows phosphatase activity towards the autophosphorylated Wzc protein, which induces colanic acid biosynthesis; catalyzes the phosphorylation of UDP-glucose dehydrogenase, an enzyme involved in colanic acid biosynthesis.
BI322_RS06915	555549/557714	WP_082236972.1	tyrosine-protein kinase (Wzc)	78	Wzc catalyzes the autophosphorylation on tyrosine residues which downregulates the biosynthesis of colonic acid (an extracellular polysaccharide).
BI322_RS06920	557836/559263	WP_082236973.1	undecaprenyl-phosphate galactose phosphotransferase (WbaP)	0	
BI322_RS06925	559308/560219	WP_082236974.1	rhamnosyltransferase	0	
BI322_RS06930	560570/561598	WP_082236975.1	hypothetical protein	31	
BI322_RS06935	561614/562714	WP_082236976.1	EpsG family protein	36	This family of proteins are related to the EpsG protein from *Bacillus subtilis*. These proteins are likely glycosyl transferases belonging to the membrane protein GT-C clan.
BI322_RS06940	562727/563569	WP_082236977.1	hypothetical protein	44	Glycosyltransferase like family 2; Members of this family of prokaryotic proteins include putative glucosyltransferase, which are involved in bacterial capsule biosynthesis.
BI322_RS06945	563574/564449	WP_082236978.1	hypothetical protein	99	Glycosyltransferase, GT2 family, putative dTDP-rhamnosyl transferase.
BI322_RS06950	564469/565359	WP_082236979.1	hypothetical protein	0	Glycosyltransferase like family 2; Members of this family of prokaryotic proteins include putative glucosyltransferase, which are involved in bacterial capsule biosynthesis.
BI322_RS06955	565408/566238	WP_082236980.1	hypothetical protein	36	
BI322_RS06960	566247/567692	WP_082236981.1	lipopolysaccharide biosynthesis protein (Wzx)	50	Membrane protein involved in the export of O-antigen and teichoic acid.
BI322_RS06965	568041/569447	WP_082236982.1	NADP-dependent phosphogluconate dehydrogenase (Gnd)	99	6-phosphogluconate dehydrogenase.
BI322_RS06970	569654/570718	WP_082236983.1	dTDP-glucose 4,6-dehydratase	99	
BI322_RS06975	570732/571601	WP_049099678.1	glucose-1-phosphate thymidylyltransferase	99	
BI322_RS06980	571633/572523	WP_082236984.1	dTDP-4-dehydrorhamnose reductase	99	
BI322_RS06985	572539/573093	WP_014230057.1	dTDP-4-dehydrorhamnose 3,5-epimerase	99	
BI322_RS06990	573267/574433	WP_082236985.1	UDP-glucose 6-dehydrogenase (Ugd)	99	
BI322_RS06995	57599/576603	WP_082236986.1	NAD-dependent epimerase	99	

^a^with the products of *K. oxytoca* strain homologues

**Table 3:** The *cps2* gene cluster of *K. oxytoca* DSM 29614

Gene (Locus tag)	Start/Stop nucleotides	Protein			
		id	Function	% of Identity^a^	Comment
BI322_RS19465	3164958/3166361	WP_004133794.1	undecaprenyl-phosphate glucose phosphotransferase (WcaJ)	100	
BI322_RS19470	3166351/3167637	WP_004133795.1	capsular biosynthesis protein (CpsB)	100	
BI322_RS19475	3167634/3168188	WP_024359125.1	capsular biosynthesis protein (CpsC)	100	Periplasmic protein involved in polysaccharide export.
BI322_RS19480	3168190/3170277	WP_004133800.1	capsular polysaccharide biosynthesis protein	100	
BI322_RS19485	3170283/3171500	WP_004133805.1	capsular polysaccharide biosynthesis protein	99	
BI322_RS19490	3171491/3172873	WP_004133807.1	hypothetical protein	100	
BI322_RS19495	3172866/3173891	WP_004133808.1	family 2 glycosyl transferase	100	
BI322_RS19500	3173872/3175086	WP_024359127.1	glycosyltransferase family 1 protein	99	
BI322_RS19505	3175083/3176198	WP_004133810.1	glycosyltransferase family 1 protein	100	
BI322_RS19510	3176195/3176605	WP_004133811.1	hypothetical protein	100	
BI322_RS19515	3176608/3178026	WP_082237571.1	DUF4832 domain-containing protein	100	
BI322_RS19520	3178029/3178535	WP_004133814.1	colanic acid biosynthesis acetyltransferase (WcaB)	100	Serine acetyltransferase involved in the biosynthesis of colanic acid, an exopolysaccharide expressed in Enterobacteraceae species.

^a^with the products of *K. oxytoca* strain homologues

In addition to tolerance to high levels of iron, the DSM 29614 strain possesses a high level of tolerance to diverse metals such as Pd, Rh, Cd, Pb, Zn, As, Hg and Ag as already mentioned. Therefore, in order to detect genetic elements putatively responsible for this capability, BacMet database [36] was used to scan the DSM 29614 strain genome. This investigation allowed to detect 149 genes putatively responsible for specific resistance to a total of 23 different kinds of metals (Fig. 6; Additional file 3: Table S3 and Additional file 5: Supporting Information).

### Phosphatase activity and metal cation precipitation

**Fig. 7:**
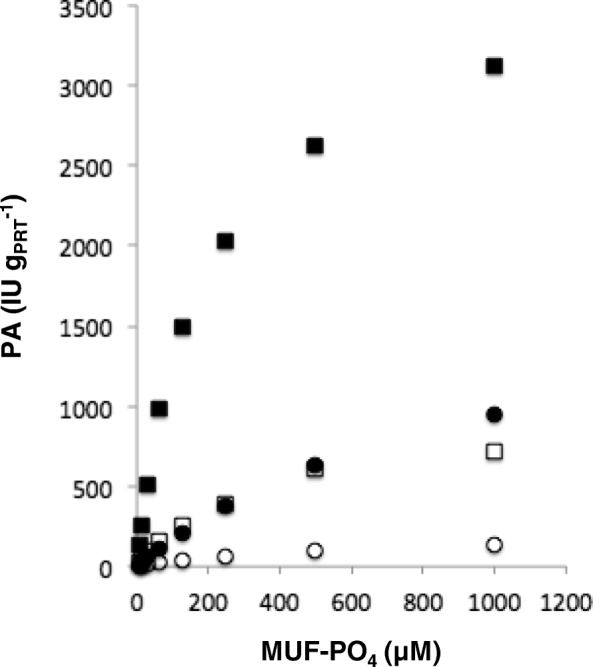
Rate of phosphatase activity (PA), reported as IU *per* g of cell proteins, in whole cells of *K. oxytoca* DSM 29614 at different concentrations of the MUF-PO_4_ fluorescence substrate after: anaerobic (solid squares) and aerobic (empty squares) cultivation in NaC medium; anaerobic (solid circles) and aerobic (empty circles) cultivation in FeC medium

**Fig. 8:**
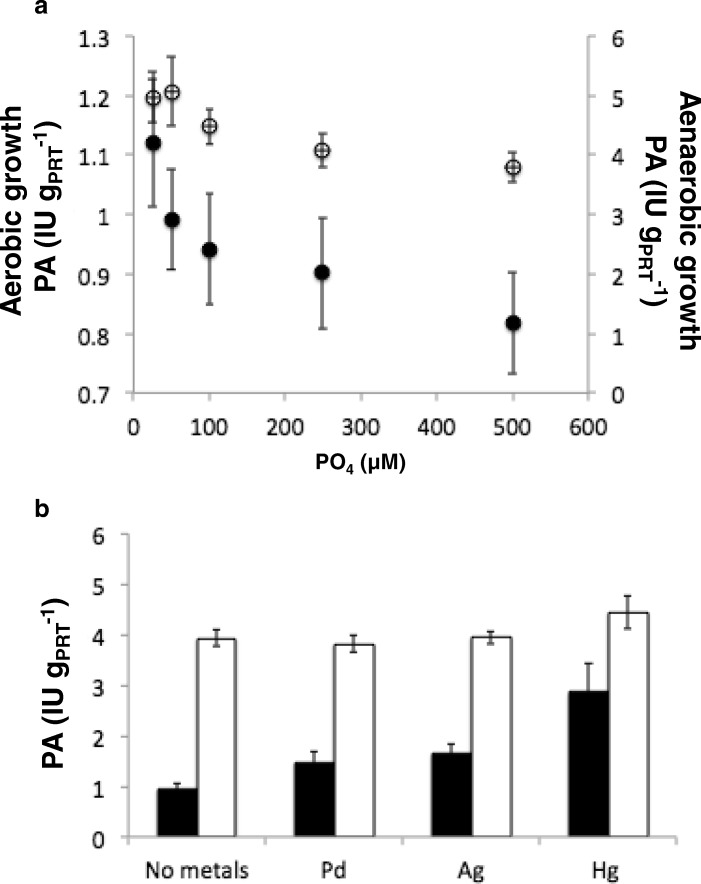
**a** Inhibition of phosphatase activity (PA), reported as IU *per* g of cell proteins, by adding different amount of PO_4_^−3^ in NaC medium under aerobic (solid circles) and anaerobic (empty circles) condition. **b** Determination of PA in NaC medium with Ag^+^, Hg^2+^ and Pd^2+^ additions (50 mg.l^− 1^) under aerobic (solid histograms) and anaerobic condition (empty histograms). Standard deviation values are reported as vertical bars

*K. oxytoca* DSM 29614 genome contains typical *pho* regulon genes [50] - like *phoA*, *phoR*, *phoB*, *phoE* and *pstSABC* – and acid phosphatases – including a phosphatase PAP2 family protein which is part of *cps1* gene cluster. The overall phosphatase activity (oPA) of DSM 29614 strain was investigated using samples collected from four different experimental sets: cultivations using FeC or in NaC media under anaerobic or aerobic conditions (Fig. 7). The highest oPA was observed in the NaC medium under anaerobic condition while the lowest oPA was revealed in FeC medium under aerobic condition. The V_max_ and K_m_ of oPA in cells cultivated under anaerobic conditions in NaC medium are by far the highest in respect to the other growing conditions (Table 4). The addition of increasing amounts of inorganic phosphate to NaC medium causes a modest but appreciable oPA decrement only in anaerobic condition, with a maximum reduction of approximately 22%comparing 25 and 500 μM (Fig. 8a). On the contrary, the addition of heavy metals to NaC growth medium strongly stimulates oPA in aerobiosis only (Fig. 8b). Indeed, the formation of struvite (MgNH_4_PO_4_.6H_2_O) was revealed in *K. oxytoca* DSM 29614 cultivations performed using NaC growth medium under aerobic conditions with the addition of different heavy metals. In particular, the addition of 50 mg.l^− 1^ Hg^2+^ or Ag^+^ induced the formation of millimetric struvite crystals (Additional file 4: Figure S2 A and B) as well as the addition of Pd^2+^ induced the formation micrometric struvite crystals (Additional file 4: Figure S2 C and D). The presence of Ag^+^ on struvite crystal surface, inferred from crystal face darkening, was confirmed by the spectrophotometric analysis revealing a content of 0.03 ± 0.01% (*w*/w).

**Table 4:** Phosphatase activity with enzymatic kinetics parameters

Growth medium	Condition	V_max_ (IU∙g_PRT_^−1^)		K_m_ (μM)	
FeC	Aerobic	0.19	±0.055	452	±30
	Anaerobic	0.73	±0.102	204	±87
NaC	Aerobic	0.83	±0.059	356	±75
	Anaerobic	4.02	±0.271	223	±15

## Discussion

The *K. oxytoca* DSM 29614 genome has peculiar characteristics which reveal a complex phylogenetic history of this microorganism. The results of the comparative genomic analysis performed using 85 *K. oxytoca* strains are in agreement with the comparative analysis of *K. pneumoniae* strain genomes [51] highlighting the enormous capacity of the strains belonging to *Klebsiella* genus to acquire (and use) exogenous DNA. Indeed, such genetic variability is typical of those groups of microorganisms that usually inhabit different niches and/or living in large (and complex) communities. Accordingly, members of *Klebsiella* genus are able to colonize diverse environments and to adapt to a range of lifestyle [52, 53]. In particular, the relative abundancies of COG categories inferred from *K. oxytoca* DSM 29614 genome could be ascribed to the necessity to survive in hazardous and metabolically demanding environments, like that in which the strain has been isolated. Accordingly, the DSM 29614 strain possesses the unique capability among the *K. oxytoca* members of growing using Fe(III)-citrate as the sole carbon and energy source under anaerobic conditions. This growth is characterized by the production of acetic acid - which is due to the involvement of CitEF, OadA, PFL, PTA and ACK during citrate anaerobic utilization [42] – and by the peculiar iron precipitation in the bacterial EPS - manly as nano-sized iron oxides and hydroxides [22] - with the concomitant massive reduction of Fe^3+^ to Fe^2+^. In general, ferric reducing bacteria, like the Gram-negative *Shewanella* and *Geobacter* spp., reduce Fe(III) to Fe(II) during the anaerobic respiration using Fe(III) as electron acceptor in a dissimilatory pathway [44–46]. As inferred from genome analysis, in the DSM 29614 strain, that does not possess genes orthologous to *Shewanella* and *Geobacter* genes involved dissimilatory pathways, cellular mechanisms controlling iron homeostasis may be taken into account for the massive reduction of Fe(III) to Fe(II), including the possible role of i) Flavin metal reductases, ii) specific efflux pumps and iii) siderophores. In addition, a direct contribution to Fe(II) production is to be ascribed to the peculiar EPS produced by DSM 29614 strain. In fact, more in general and in agreement with what described by Gupta et al. (2017) [54], the resistance of *K. oxytoca* DSM 29614 to different metals could be due to the synthesis of the EPS, which promotes metal cation entrapment and reduction with the production of metal NPs which finally resulted embedded into EPS matrix itself [10, 15–22, 25].

One of the two *cps* clusters identified in *K. oxytoca* DSM 29614 genome, namely *cps1*, could be responsible for the biosynthesis of DSM 29614 EPS that was characterized by Leone et al. (2007) [11]. In fact, one of the most peculiar characteristics of this EPS is the presence of the rare sugar rhamnose and *cps1* contains genes encoding a rhamnosyltransferase (BI322_RS06925) and two proteins involved in rhamnose metabolism (BI322_RS06980 and BI322_RS06985). In addition, the presence of *his* genes associated with *cps1* is noteworthy as well (Additional file 6: Table S4 and Additional file 5: Supporting Information), since histidine was observed as a major amino acid component of protein fraction associated with DSM 29614 strain EPS [14]. Histidine plays a role in metal homeostasis acting either as metal chelator free-amino acid and as metal-coordinating residues in proteins, like metal chaperones or histidine-rich proteins [55–59].

In addition, genes whose products are putatively involved in general and/or metal-specific resistance processes were detected in DSM 29614 genome. In particular, 149 genes were identified, globally accounting for specific resistance to 23 different kinds of metals. On the other hand, as an example of general metal-detoxifying strategies, Fe-SOD - contrasting the intracellular generation of superoxide - was so far shown accumulating in presence of Fe(III) during both anaerobic and aerobic growth (Gallo et al., 2012) [26] (Additional file 2: Table S2). Furthermore, phosphate salt precipitation as struvite [60] can play a role in general processes devoted to metal detoxification in *K. oxytoca* DSM 29614 as it can be inferred from TEM observations and from the oPA profile in NaC and FeC medium according to Yung et al. (2014) [61] and Montgomery et al. (1995) [62]. The struvite was obtained in *K. oxytoca* DSM 29614 cultivations only under aerobic conditions because of favourable pH conditions (around 8; Fig. 5a) [63]. Thus, NH4^+^, Mg2^+^, PO4^3−^ and other cations, embedded in EPS, reach the critical supersaturation conditions typical of a nucleation site. Conversely, at pH < 7 struvite does not form, although high concentrations of PO4^3−^ remains sequestered by EPS. Indeed, it has been demonstrated by EDS-SEM element-maps of Pd-Fe NPs that Pd was associated with P but not with Fe [20]. The finding that the addition of toxic heavy metals to NaC medium strongly stimulates oPA activity only in aerobiosis (Fig. 8b) suggest that heavy metal cations can be embedded into EPS which is massively produced in anaerobiosis while they can form metal ions-phosphate salts in aerobiosis. Thus, the different effect on oPA, observed comparing aerobic and anaerobic conditions upon heavy metal additions to cultivation medium, suggests an interplay between inorganic phosphate and EPS biosynthesis that may have consequences on metal resistance of *K. oxytoca* DSM 29614. In this context, it was previously observed in a phosphatase-overproducing *Citrobacter* sp. (NCIMB 40259) grown in a bioreactor an interesting correlation between i) limitation of carbon, phosphorus or nitrogen, ii) the capability of liberating inorganic phosphate from an organic phosphate donor with the precipitation of metal cations as insoluble salt at the cell surface, and iii) biofilm formation [64]. In addition, a clear correlation between inorganic phosphate limitation and EPS production was observed in *Mycobacteria* [65].

## Conclusion

The *K. oxytoca* DSM 29614 genome shows peculiar characteristics which confirm the view of an open pangenome for *Klebsiella* species which is in agreement with the wide distribution of member of *Klebsiella* genus. The *K. oxytoca* DSM 29614 unique capabilities of using Fe(III)-citrate as sole carbon and energy source in anaerobiosis and tolerating diverse metals coincides with the presence at the genomic level of specific genes that can support i) energy metabolism optimization, ii) cell protection by the biosynthesis of a peculiar exopolysaccharide armour entrapping metal ions and iii) general and metal-specific detoxifying activities by different proteins and metabolites. Therefore, *K. oxytoca* DSM 29614 strain can play a role in nano-biotechnological applications and in the study of bacterial adaptation to hazardous and metabolically demanding environments.

## Additional files

Additional file 1: **Table S1.** List of genomes of *K. oxytoca* strains used for comparative genomics. (XLSX 10 kb)

Additional file 2: **Table S2.** List of differentially abundant proteins in DSM 29614 strain used for protein functional enrichment analysis. (XLSX 17 kb)

Additional file 3: **Table S3.** List of metal resistance genes identified in the DSM 29614 strain genome. (XLSX 48 kb)

Additional file 4: **Figure S1.** *K. oxytoca* DSM 29614 grown in NaC (left) and in FeC medium (right) after 7 day of incubation at 30 °C. **Figure S2.** a,b) Production of ortorhomboidal struvite in the presence of Hg^2+^ (a) and Ag^+^ (b); both crystals precipitated in aerobic cultures of *K. oxytoca* DSM 29614. c,d) Micrographs of microcrystal of struvite by *K. oxytoca* DSM 29614 cells, grown in aerobic conditions in the presence of Pd^2+^ in transmission mode (c) and in fluorescence mode (d) with cells stained with DAPI. e) Micrographs of TEM of microcrystals of struvite coated by Pd^2+^. (PDF 527 kb)

Additional file 5: Supporting Information. (PDF 130 kb)

Additional file 6: **Table S4.** List of genes associate with *cps1* cluster in DSM 29614 strain. (XLSX 10 kb)

## Abbreviations

Ag: Silver
As: Arsenic
Cd: Cadmium
*cps*: Capsule biosynthetic gene cluster
DAPI: 4′,6-diamidino-2-phenylindole
EPS: Exopolysaccharide
Fe: Iron
FeC: Growth medium containing 50 mM Fe(III)-citrate
FEPCU: Fisher Exact Parent-Child Union
Hg: Mercury
MUF-PO_4_: 4-methylumbelliferyl phosphate disodium salt
NaC: Growth medium containing 50 mM Na-citrate
NPs: Nanoparticles
PA: Phosphatase activity
Pb: Lead
Pd: Palladium
Rh: Rhodium
SOD: Superoxide dismutase
Zn: Zinc
