# Supplementary material for: Genomic traits of Klebsiella oxytoca DSM 29614, an uncommon metal-nanoparticle producer strain isolated from acid mine drainages
Source: BMC Microbiol. 2018 Nov 27;18:198. doi: 10.1186/s12866-018-1330-5 (PMC6258164; doi:10.1186/s12866-018-1330-5)
Supplement: Supplementary file 4 — Figure S1. K. oxytoca DSM 29614 grown in NaC (left) and in FeC medium (right) after 7 day of incubation at 30 °C. Figure S2. a,b) Production of ortorhomboidal struvite in the presence of Hg2+ (a) and Ag+ (b); both crystals precipitated in aerobic cultures of K. oxytoca DSM 29614. c,d) Micrographs of microcrystal of struvite by K. oxytoca DSM 29614 cells, grown in aerobic conditions in the presence of Pd2+ in transmission mode (c) and in fluorescence mode (d) with cells stained with DAPI. e) Micrographs of TEM of microcrystals of struvite coated by Pd2+. (PDF 527 kb) [file 12866_2018_1330_MOESM4_ESM.pdf]

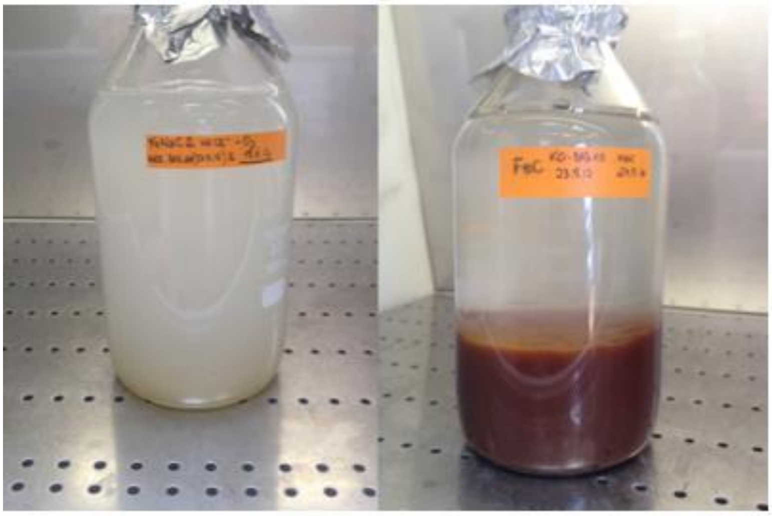

Figure S1. *K. oxytoca* DSM 29614 grown in NaC (left) and in FeC medium (right) after 7 day of incubation at 30 °C.

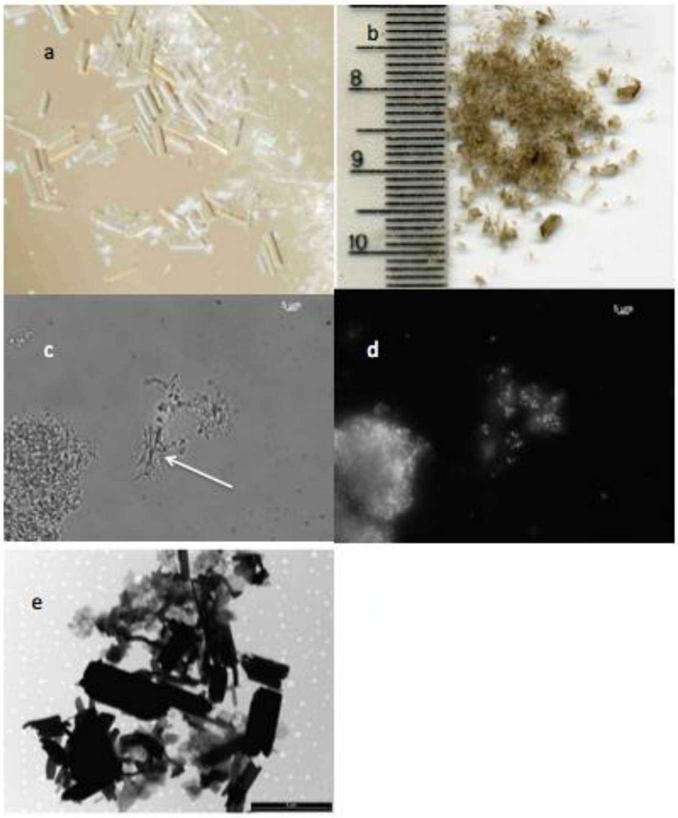

Figure S2. a,b)Production of ortorhomboidal struvite in the presence of Hg^{2+} (a) and Ag^{+} (b); both crystals precipitated in aerobic cultures of *K. oxytoca* DSM 29614. c,d) Micrographs of microcrystal of struvite by *K. oxytoca* DSM 29614 cells, grown in aerobic conditions in the presence of Pd^{2+} in transmission mode (c) and in fluorescence mode (d) with cells stained with DAPI. e) Micrographs of TEM of microcrystals of struvite coated by Pd^{2+}.
