# Supplementary material for: Genomic traits of Klebsiella oxytoca DSM 29614, an uncommon metal-nanoparticle producer strain isolated from acid mine drainages
Source: BMC Microbiol. 2018 Nov 27;18:198. doi: 10.1186/s12866-018-1330-5 (PMC6258164; doi:10.1186/s12866-018-1330-5)
Supplement: Supplementary file 5 — Supporting Information. (PDF 130 kb) [file 12866_2018_1330_MOESM5_ESM.pdf]

Genomic traits of *Klebsiella oxytoca* DSM 29614, an uncommon metal-nanoparticle producer strain isolated from acid mine drainages

Giuseppe Gallo[1*], Luana Presta[2*], Elena Perrin[2], Michele Gallo[3], Davide Marchetto[3], Anna Maria Puglia[1], Renato Fani[2], Franco Baldi[3]

Affiliations:

[1]Laboratory of Molecular Microbiology and Biotechnology, Dept. of Biological, Chemical and Pharmaceutical Sciences and Technologies, Viale delle Scienze, ed. 16, 90128, Palermo.

[2]Laboratory of Microbial and Molecular Evolution, Dept of Biology, University of Florence, Via Madonna del Piano 6, I-50019 Sesto F.no (Florence), Italy.

[3]Dipartimento di Scienze Molecolari e Nanosistemi, University Cà Foscari Venezia, Via Torino 155. 30172 Mestre Venezia (Italy).

[*]Authors contribuiting to the work equally.

Corresponding author: Giuseppe Gallo

Supporting Information

Insights on *cps1* and *cps2* gene clusters identified in the DSM 29614 strain genome

The *cps1* is part of a 47.412 Kb region containing a total of 41 ORFs (from BI322_RS06885 to BI322_ RS07085) identified by an AntiSmash analysis. The *cps1* gene cluster comprises 23 genes (from BI322_RS06885 to BI322_RS06995) which are defined by genes encoding GalF, the traslocon Wzi, Wza, Wzb, Wzc and the initial glycosyltransferase undecaprenyl-phosphate galactose phosphotransferase (WbaP), from one hand, and Gnd and Ugd, from the other one, as well as it is usually reported for *Klebsiella* spp. [1,2]. Indeed, 9 out 23 *cps1* gene products show low (from 50% to 31%) or no sequence identity with the products of *K. oxytoca* homologous genes further confirming the peculiarity of DSM 29614 strain. It is interesting to note that, in agreement with the observations by Folladore *et al.* (2016) [3], the *cps1* genes are located in association with *rfb* (from BI322_RS07000 to BI322_RS07030) and *his* genes (from BI322_RS07035 to BI322_RS07070) required for the O-antigen (i.e. the outermost part of the lipopolysaccharide) and histidine biosynthesis, respectively (Table S4, Additional files). The flanking regions of *cps1-rfb-his* locus are also worth of mention since they contain, from one hand, a gene encoding a TerC family protein (BI322_ RS06880), which is known to be involved in heavy metal tolerance [4] and, from the other one, a region containing *nif* genes (from BI322_RS07090 to) devoted to nitrogen fixation, respectively. On the other hand, the *cps2* gene cluster spans on a 13.577 Kb region containing 11 ORFs (from BI322_RS19465 to BI322_RS19520), which are mainly involved in biosynthesis, transfer and export of exopolysaccharides. Unlike *cps1*, the presence of genes encoding WcaJ (BI322_RS19465) and WcaB (BI322_RS19520) characterizes *cps2* cluster. This gene cluster, whose products are highly conserved among *K. oxytoca* strain, is flanked by a region containing genes (from BI322_RS19430 to BI322_RS19440) involved in biosynthesis of glucuronate and a gene (BI322_RS19450) encoding FoxA, a ferrioxamine receptor.

Insights on metal resistance genes identified in the DSM 29614 strain genome

The DSM 29614 strain possesses a high level of tolerance to heavy metals like Au, Ag, As, Cd, Co, Cr, Cu, Ni, Hg, Pb, Pd, Pt, Rh, Ti, Se, Zn. The BacMet database [5] was used to scan the genome of DSM 29614 strain highlighting genetic elements putatively responsible for resistance to metals. More precisely, several genes were found that could be responsible for Zn resistance, which might be conferred by the set of *czc* genes [6,7] that usually impart cross-resistance also to Co and Cd. This specific mechanism is usually related to a cation transport pump located in the inner membrane and encoded by *czcABC* genes. The strain apparently possesses only *czcA*, whose product is the basic inner membrane transport protein. In addition, *czcD* (whose product may act as a membrane-bound sensor protein or as a metal ion transporter) and *czcP* (that usually enhance metal resistance relying on the action of some ATPases encoded by *zntA*, *cadA*, *pbrA*) [8] were alsodetected. The two component regulatory systems *czcS/czcR* [9] involved into Zn homeostasis mechanisms were revealed too. Zn resistance in DSM 29614 strain could also be related to the detected *zntA* [10] transport system (and its transcriptional regulator *zntR*) and to *cadACRX* genes cluster [11]: the first encodes an ATPase, which usually leads to Cd and Hg resistance; the second codes for an ATPase very efficient for Cd transport, but which carries Zn and Co too. Both systems may then be responsible for two of the three metal resistance observed, i.e. Cd and Zn. Pb resistance could rely on the presence on the *K. oxytoca* DSM 29614 genome of *pbrABC* genes [12], which combine functions involved in uptake, efflux, and accumulation of Pb(II), and its regulatory system *pbrR.* By the way, these are only the main systems generally accounted to confer Zn, Cd and Pb resistances. Indeed, many other genes may have a role when dealing with such phenotypes (Table S3, Additional files). Data reported in Table S3 (Supplementary Material) highlight a possible role of genes accounting for the capability of *K. oxytoca* DSM

29614 to grow also in the presence of Ag (*silABCEFPRS* genes) [7], Cu (*copABDRSZ* genes) [13], As *arsABCDMR* [14], Ni *nccABH* [15], Cr (*chrCR*) [16] and Hg (*merA, merR2*) [17].

References

1. Shu H-Y, Fung C-P, Liu Y-M, Wu K-M, Chen Y-T, Li L-H, et al. Genetic diversity of capsular polysaccharide biosynthesis in *Klebsiella pneumoniae* clinical isolates. Microbiology. England; 2009;155:4170–83.
2. Pan Y-J, Lin T-L, Chen C-T, Chen Y-Y, Hsieh P-F, Hsu C-R, et al. Genetic analysis of capsular polysaccharide synthesis gene clusters in 79 capsular types of *Klebsiella* spp. Sci Rep. 2015;5:15573.
3. Follador R, Heinz E, Wyres KL, Ellington MJ, Kowarik M, Holt KE, et al. The diversity of *Klebsiella pneumoniae* surface polysaccharides. Microb Genomics . 2016; 2:e000073.
4. Burian J, Tu N, Kl'ucar L, Guller L, Lloyd-Jones G, Stuchlik S, et al. In vivo and in vitro cloning and phenotype characterization of tellurite resistance determinant conferred by plasmid pTE53 of a clinical isolate of *Escherichia coli*. Folia Microbiol; 1998;43:589–99.
5. Pal C, Bengtsson-Palme J, Rensing C, Kristiansson E, Larsson DGJ. BacMet: antibacterial biocide and metal resistance genes database. Nucleic Acids Res. 2014;42:D737–43.
6. Nies DH. CzcR and CzcD, gene products affecting regulation of resistance to cobalt, zinc, and cadmium (*czc* system) in *Alcaligenes eutrophus*. J Bacteriol. 1992;174:8102–10.
7. Silver S, Gupta A, Matsui K, Lo JF. Resistance to Ag(i) cations in bacteria: environments, genes and proteins. Met Based Drugs. 1999;6:315–20.
8. Scherer J, Nies DH. CzcP is a novel efflux system contributing to transition metal resistance in *Cupriavidus metallidurans* CH34. Mol Microbiol. 2009;73:601–21.
9. van der Lelie D, Schwuchow T, Schwidetzky U, Wuertz S, Baeyens W, Mergeay M, et al. Two-component regulatory system involved in transcriptional control of heavy-metal homoeostasis in *Alcaligenes eutrophus*. Mol Microbiol. England; 1997;23:493–503.

10. Rensing C, Mitra B, Rosen BP. The *zntA* gene of *Escherichia coli* encodes a Zn(II)-translocating P-type ATPase. Proc Natl Acad Sci U S A. 1997;94:14326–31.
11. Lee S-W, Glickmann E, Cooksey DA. Chromosomal Locus for cadmium resistance in *Pseudomonas putida* consisting of a cadmium-transporting ATPase and a MerR family response regulator. Appl Environ Microbiol. 2001;67:1437–44.
12. Borremans B, Hobman JL, Provoost A, Brown NL, van Der Lelie D. Cloning and functional analysis of the *pbr* lead resistance determinant of *Ralstonia metallidurans* CH34. J Bacteriol. 2001;183:5651–8.
13. Adaikkalam V, Swarup S. Characterization of *copABCD* operon from a copper-sensitive *Pseudomonas putida* strain. Can J Microbiol. 2005;51:209–16.
14. Suzuki K, Wakao N, Kimura T, Sakka K, Ohmiya K. Expression and regulation of the arsenic resistance operon of *Acidiphilium multivorum* AIU 301 plasmid pKW301 in *Escherichia coli*. Appl Environ Microbiol. 1998;64:411–8.
15. Schmidt T, Schlegel HG. Combined nickel-cobalt-cadmium resistance encoded by the *ncc* locus of *Alcaligenes xylosoxidans* 31A. J Bacteriol. 1994;176:7045–54.
16. Branco R, Chung AP, Johnston T, Gurel V, Morais P, Zhitkovich A. The chromate-inducible *chrBACF* operon from the transposable element Tn*OtChr* confers resistance to chromium(VI) and superoxide. J Bacteriol. 2008;190:6996–7003.
17. Gupta A, Phung LT, Chakravarty L, Silver S. mercury resistance in *Bacillus cereus* RC607: transcriptional organization and two new open reading frames. J Bacteriol 1999;181:7080–6.
